# *EGFR* gene copy number as a predictive biomarker for the treatment of metastatic colorectal cancer with anti-EGFR monoclonal antibodies: a meta-analysis

**DOI:** 10.1186/1756-8722-5-52

**Published:** 2012-08-16

**Authors:** Zu-Yao Yang, Wei-Xi Shen, Xue-Feng Hu, Da-Yong Zheng, Xin-Yin Wu, Ya-Fang Huang, Jin-Zhang Chen, Chen Mao, Jin-Ling Tang

**Affiliations:** 1 Division of Epidemiology, the Jockey Club School of Public Health and Primary Care, the Chinese University of Hong Kong, Hong Kong, People’s Republic of China; 2 Cancer Institute, the Second Clinical Medical College, Jinan University, Shenzhen People's Hospital, Shenzhen, Guangdong Province, People’s Republic of China; 3 Department of Oncology, Nanfang Hospital, Southern Medical University, Guangzhou, Guangdong Province, People’s Republic of China

**Keywords:** Colorectal neoplasms, Monoclonal antibodies, Cetuximab, Panitumumab, Epidermal growth factor receptor, Systematic review, Meta-analysis

## Abstract

**Background:**

Epidermal growth factor receptor gene copy number (*EGFR* GCN) has been heavily investigated as a potential predictive biomarker for the treatment of metastatic colorectal cancer (mCRC) with anti-EGFR monoclonal antibodies (MAbs). The objective of this study was to systematically review current evidences on this issue.

**Methods:**

PubMed, EMBASE, The Cochrane Library, Chinese Biomedical Literature Database, Wanfang Data, and the conference abstracts of American Society of Clinical Oncology and European Society of Medical Oncology were comprehensively searched. Studies that reported the objective response rate (ORR), progression-free survival, and/or overall survival of mCRC patients treated with anti-EGFR MAbs, stratified by *EGFR* GCN status, were included. The effect measures for binary outcome (response) and time-to-event outcomes (progression-free survival and overall survival) were risk difference and hazard ratio, respectively. Statistical heterogeneity among the studies was assessed by the Cochran’s *Q*-test and the *I*^2^ statistic. If appropriate, a quantitative synthesis of data from different studies would be conducted with a random-effects model.

**Results:**

Nineteen eligible studies were identified. The criteria for increased *EGFR* GCN (GCN+) were highly inconsistent across different studies. The prevalence of GCN + ranged from 6.9% to 88.9%, and the difference in ORR between patients with GCN + and those with non-increased *EGFR* GCN (GCN-) varied from −28% to 84%. Because of the significant heterogeneity, no quantitative synthesis of data was performed. There was a general trend towards higher ORR in patients with GCN+. The difference in ORRs between patients with GCN + and those with GCN- was even greater in *KRAS* wild-type patients, while in *KRAS* mutated patients the difference often did not exist. Almost all patients with *EGFR* amplification responded to the treatment. However, the prevalence of *EGFR* amplification was generally low. Incomplete data on progression-free survival and overall survival seemingly supported the findings on ORR.

**Conclusions:**

Although increased *EGFR* GCN is generally associated with a better outcome of anti-EGFR MAbs treatment, especially among patients with wild-type *KRAS*, the clinical utility of this biomarker for selecting recipients of anti-EGFR MAbs would be severely limited by the heterogeneous scoring system and the poor reproducibility of *EGFR* GCN enumeration due to technical reasons.

## Background

Colorectal cancer (CRC) is the third most common malignant disease and the fourth leading cause of cancer-related deaths worldwide [1]. Synchronous metastases have occurred in about 25% of patients at the time of diagnosis, and an additional 40% to 50% develop secondary metastases during the course of their disease after diagnosis [2]. For most patients with metastatic CRC (mCRC), chemotherapy is traditionally the first choice. However, the response rate is usually less than 50% [2] and the five-year survival rate of mCRC patients remains below 10% [3].

The chimeric IgG1 cetuximab and the fully humanized IgG2 panitumumab, two monoclonal antibodies (MAbs) targeted at epidermal growth factor receptor (EGFR), were found effective in combination with chemotherapy or as a single agent for the treatment of chemotherapy-resistant mCRC [4-6]. However, the tumor response rate increased by anti-EGFR MAbs was only 10%-20%, whether it be used as the 1^st^- or 2^nd^-line treatment [4,6-9]. As anti-EGFR MAbs were associated with significant increase in toxicities [5] and costs [10], it is important to identify the responsive patients for treatment and prevent non-responsive ones from exposure to unnecessary treatment.

It has been established that *KRAS* mutations are a strong predictor of resistance to anti-EGFR MAbs [11-13]. However, a significant proportion of patients with wild-type *KRAS* remain unresponsive to anti-EGFR MAbs. Therefore, the identification of new biomarkers that can be used jointly with *KRAS* has become appealing in predicting treatment response.

Moroni and colleagues reported for the first time a strong relation between *EGFR* gene copy number (GCN) and the response of patients to anti-EGFR MAbs [14]. This relation has since been substantially investigated. However, published studies on this topic are generally small in sample size, which may have led to inconsistent results, and thus each study alone may not be strong enough to produce a firm conclusion [15]. In addition, sparse data from individual studies is available to assess the impact of *EGFR* GCN on such patient-important outcomes as progression-free survival (PFS) and overall survival (OS) [16,17].

Therefore, we conducted a systematic review of current evidences to assess the predictive role of an increase of *EGFR* GCN in the treatment of mCRC with anti-EGFR MAbs, with a hope to take a step further towards the ultimate end of personalized treatment of mCRC.

## Results

**Figure 1:**
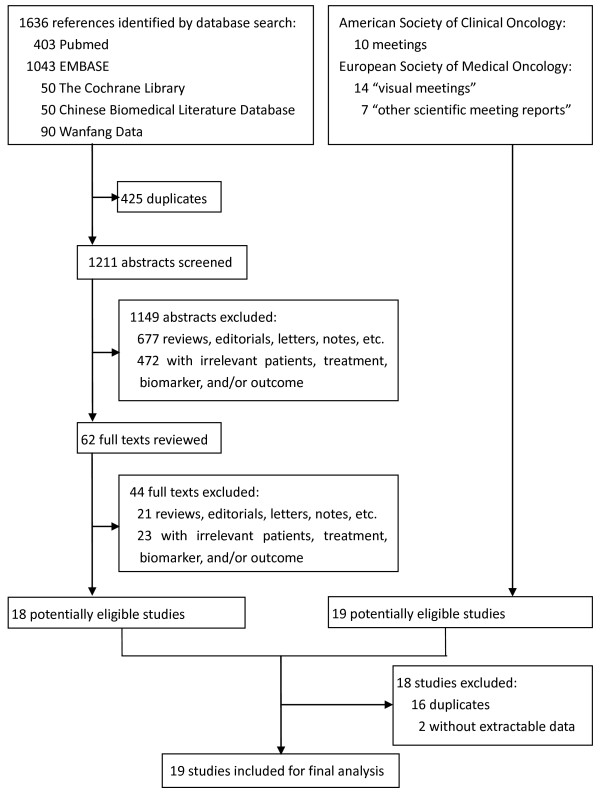
Flow chart of study selection.

Figure 1 shows the inclusion and exclusion of studies step by step. In total, 19 eligible studies were identified [14-32], of which 17 provided data on ORR [14-26,28-31] and 15 on PFS or OS [15-21,24,25,27-32].

### Description of the studies

The basic characteristics of these studies were summarized in Table 1. Most of them were retrospective studies, with sample sizes varying from 27 to 155. Three studies were conducted in *KRAS* wild-type patients only [16,31,32], and another eight studies reported the data on *KRAS* wild-type and mutant patients separately [14,18,19,22,24-26,28], providing us the opportunity to examine the impact of *KRAS* status on the predictive power of GCN+. The anti-EGFR MAb administered, the response criteria, and the assay for *EGFR* GCN quantification were generally consistent across different studies. However, the lines of treatment and the sources of tumor samples used for GCN testing were relatively inconsistent.

**Table 1:** Basic characteristics of the 19 eligible studies

**Study**	**Design**	** *N* **	** *KRAS* mutation**	**Agent; treatment modality**	**Line**	**Response criteria**	**Tumor sample**	**GCN assay**
Bengala 2009^25^	Retrospective	55	36.5 %	Cmab/Pmab; CT	NA	NA	NA	FISH
Campanella 2010^26^	Retrospective	88	37.7 %	Cmab; mixed	Mixed	RECIST	NA	FISH
Cappuzzo 2008^23^	Retrospective	85	NA	Cmab; CT	≥2nd	RECIST	Mixed	FISH
Frattini 2007^27^	Retrospective	27	37.0 %	Cmab; CT	Mixed	RECIST	Primary	FISH
Gevorgyan 2007^28^	Retrospective	40	NA	Cmab; CT	NA	NA	Primary	FISH
Goncalves 2008^29^	Retrospective	29	44.8 %	Cmab; CT	≥2nd	WHO	Mixed	FISH
Italiano 2008^15^	Retrospective	41	NA	Cmab; CT	NA	RECIST	Primary	FISH
Khambata-Ford 2007^30^	Prospective	56	34.6 %	Cmab; MT	≥2nd	WHO	Metastatic	Q-PCR
Laurent-Puig 2009^16^	Retrospective	96	0 %	Cmab; CT	≥2nd	RECIST	Mixed	FISH + CISH
Lievre 2006^31^	Retrospective	30	43.3 %	Cmab; CT	≥2nd	RECIST	Primary	CISH
Mancuso 2008^32^	Retrospective	31	32.3 %	Cmab; NA	NA	NA	NA	FISH
Moroni 2005^14^	Retrospective	29	31.0 %	Cmab/Pmab; mixed	Mixed	RECIST	Primary	FISH
Perrone 2009^24^	Retrospective	31	24.1 %	Cmab; CT	≥2nd	RECIST	Mixed	FISH
Personeni 2008^33^	Retrospective	87	33.3 %	Cmab; mixed	≥3rd	RECIST	NA	FISH
Razis 2008^34^	Retrospective	66	NA	Cmab; CT	≥2nd	NA	Metastatic	FISH
Sartore-Bianchi 2007^17^	Retrospective	58	NA	Pmab; MT	≥2nd	RECIST	NA	FISH
Sastre 2009^35^	Prospective	36	21.7 %	Cmab; MT	1st	WHO	NA	FISH
Scartozzi 2009^36^	Retrospective	37	0 %	Cmab; CT	≥2nd	RECIST	Primary	FISH
Tol 2010^37^	Retrospective	155	0 %	Cmab; CT	1st	RECIST	Primary	FISH
Study	Criteria for GCN+							GCN + %
Bengala 2009^25^	Gene copies/nucleus ≥ 2.9							30.9
Campanella 2010^26^	Polysomy: CEP7 ≥ 3; amplification: gene/CEP7 ≥ 2.0							68.2
Cappuzzo 2008^23^	Gene copies/nucleus ≥2.92*							50.6
Frattini 2007^27^	High polysomy: CEP7 > 4 in ≥ 50 % of cells; amplification: gene/CEP7 > 3 in ≥ 10 % of cells							88.9
Gevorgyan 2007^28^	High polysomy: ≥ 4 gene copies in ≥ 40 % of cells; amplification: gene clusters, gene/CEP7 ≥ 2, or ≥ 15 gene copies in ≥ 10 % of cells							20.0
Goncalves 2008^29^	High polysomy: ≥ 4 gene copies in ≥ 40 % of cells and gene/CEP7 ≤ 2; amplification: gene/CEP7 ≥ 2 or ≥ 15 gene copies in ≥ 10 % of cells							6.9
Italiano 2008^15^	High polysomy: ≥ 4 gene copies in ≥ 40 % of cells; amplification: gene/CEP7 ≥ 2, or ≥ 15 gene copies in ≥ 10 % of cells							19.5
Khambata-Ford 2007^30^	NA							7.1
Laurent-Puig 2009^16^	High polysomy: ≥ 4 gene copies in ≥ 40 % of cells; amplification: gene clusters, gene/CEP7 ≥ 2, or ≥ 10 gene copies in ≥10 % of cells							17.7
Lievre 2006^31^	Amplification: ≥ 6 gene copies in > 50 % of cells, or gene clusters							10.0
Mancuso 2008^32^	Gene copies/nucleus ≥ 3							58.1
Moroni 2005^14^	Gene copies/nucleus ≥ 3							31.0
Perrone 2009^24^	Gene copies/nucleus ≥ 2.79*							74.2
Personeni 2008^33^	Gene copies/nucleus ≥ 2.83*							37.9
Razis 2008^34^	gene/CEP7 > 1.2							7.6
Sartore-Bianchi 2007^17^	Polysomy: gene copies/nucleus ≥ 3; amplification: gene/CEP7 ≥ 2; in ≥ 43 % of cells							32.8
Sastre 2009^35^	High polysomy: ≥ 4 gene copies in ≥ 40 % of cells; amplification: gene/CEP7 ≥ 2, or ≥ 15 gene copies in ≥ 10 % of cells							30.6
Scartozzi 2009^36^	Gene copies/nucleus ≥ 2.6*							40.5
Tol 2010^37^	Gene copies/nucleus ≥ 3, or gene/CEP7 ≥ 2							15.3

*N*, sample size; GCN, gene copy number; GCN+, increased gene copy number; Cmab, cetuximab; Pmab, panitumumab; CT, combination therapy; NA, not available; RECIST, Response Evaluation Criteria in Solid Tumors; FISH, fluorescent in situ hybridization; CEP7, chromosome 7; WHO, World Health Organization criteria; MT, monotherapy; Q-PCR, quantitative polymerase chain reaction; CISH, chromogenic in situ hybridization.

* the cutoff point was identified by Receiver Operating Characteristics analysis.

Most notably, the criteria for GCN + were highly heterogeneous among different studies. In one study [26], only gene amplification was considered as GCN+. In six studies [15,16,22-24,30], GCN + included both high level of polysomy and amplification. In two studies [17,21], all levels of polysomy and amplification were considered as GCN+. The definitions of polysomy, high polysomy, and amplification, respectively, also varied across studies. The most commonly used criteria for high polysomy and amplification were “≥ 4 gene copies in ≥ 40 % of cells” [15,16,23,24,30] and “gene/CEP7 ≥ 2, or ≥ 15 gene copies in ≥ 10% of cells” [15,23,24,30], respectively. In seven studies [14,18-20,27,28,31], only the average gene copies per nucleus was used to define GCN+, with the cutoff points varying from 2.6 to 3. For two of the seven studies [14,27] where the cutoff point was “gene copies/nucleus ≥ 3”, GCN + could be viewed as polysomy according to the definition given in the study of Sartore-Bianchi et al. [17]. Similarly, the criteria used in the study of Tol et al. [32] was “gene copies/nucleus ≥3, or gene/CEP7 ≥ 2”, which could be viewed as approximate to “polysomy or amplification” according to the definitions from the studies of Campanella et al., Sartore-Bianchi et al., and others (Table 1).

The prevalence of GCN + in these studies ranged from 6.9% to 88.9%, partly reflecting the significant heterogeneity in the criteria for GCN+. Even in studies that used the same criteria to define GCN+, the prevalence of GCN + also varied considerably. For example, see the studies of Bengala et al. and Cappuzzo et al.; the studies of Gevorgyan et al., Goncalves et al., Italiano et al. and Sastre et al.; the studies of Mancuso et al. and Moroni et al.; or the studies of Perrone et al. and Personeni et al. (Table 1). As shown in Table 2, the prevalence of gene amplification was generally low, ranging from 0 to 10%, except in two studies.

**Table 2:** **Prevalence and objective response rate of patients with different statuses of *EGFR* gene**

**Study**	** *N* **	**Prevalence (positive/total)**				**Objective Response Rate (responder/total)**			
		**Amplification**	**High polysomy**	**Other GCN+**	**GCN-**	**Amplification**	**High polysomy**	**Other GCN+**	**GCN-**
Campanella 2010^26^	88	4/88		84/88		NR		NR	
Frattini 2007^27^	27	8/27	16/27			6/8	4/16	0/3	
Gevorgyan 2007^28^	40	8/40		19/40	12/40	0/8		0/19	5/13
Gonvalves 2008^29^	29	0/29	2/29	10/29	17/29	NA	2/2	3/10	4/17
Italiano 2008^15^	41	0/41	8/41	2/41	31/41	NA	2/8	8/33	
Lievre 2006^31^	30	3/30		27/30		3/3		8/27	
Moroni 2005^14^*	29	7/29	2/29		20/29	6/7	2/2		1/20
Perrone 2009^24^	31	2/31	21/31		8/31	1/2	4/21		4/8
Personeni 2008^33^	87	2/87	31/87		54/87	2/2	15/31		8/54
Sartore-Bianchi 2007^17^	58	0/58		58/58		NA			6/58
Tol 2010^37^	556	13/556	72/556		471/556	NR	NR	NR	NR

*N*, sample size; GCN+, increased gene copy number; GCN-, non-increased gene copy number; NR, not reported; NA, not applicable.

* This study did not specify the criteria for “amplification” explicitly. We applied the most popular criterion (gene/CEP7 ≥ 2, see Table 1) to categorize the patients of this study.

### The association of *EGFR* gene copy number status with clinical outcomes

**Figure 2:**
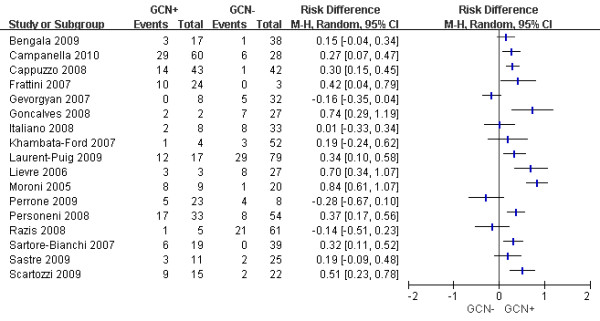
**Difference in objective response rate between GCN + and GCN- patients.** For GCN + group, the total events and patients were 125 and 301, respectively. For GCN- group, the total events and patients were 106 and 590, respectively. Heterogeneity test: *P* <0.00001, *I*^2^ = 78%.

The ORRs stratified by *EGFR* GCN status were summarized Figure 2. There was significant statistical heterogeneity among the studies (*P* < 0.00001, *I*^2^ = 78%). Even when we pooled only the studies using identical criteria for GCN+, the heterogeneity sustained. In view of this, and especially considering the heterogeneous methodological as well as clinical characteristics, we decided not to perform quantitative synthesis of the studies, for it would be clinically meaningless and the results would be difficult to interpret.

The difference in ORRs between patients with GCN + and those with GCN- varied from −28% to 84%. Visually, there was a general trend towards higher ORR in patients with GCN + (Figure 2). Five studies [14,19,22,26,28] provided data on the ORR of *EGFR* amplified patients, which also indicated a trend that the ORR increased with GCN (Table 2), although the sample sizes were too small to produce a firm conclusion. Of the 22 *EGFR* amplified patients, 18 experienced an objective response, representing an ORR of 82%. Among the four patients who did not respond, three had *KRAS* or *PIK3CA* exon 20 mutations [14,22].

**Figure 3:**
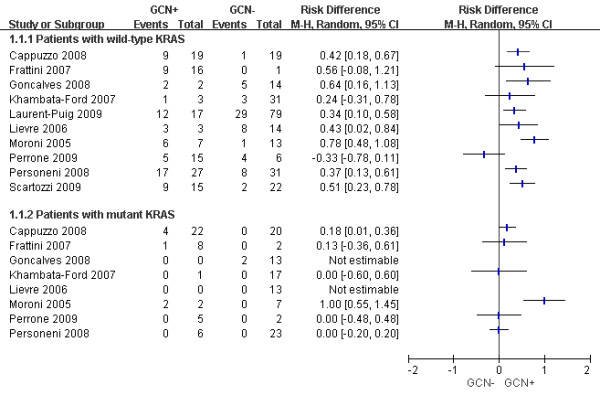
**Difference in objective response rate between GCN + and GCN- patients, stratified by *KRAS * status.** In patients with wild-type *KRAS *, the total events and patients were 73 and 124, respectively, for GCN + group, and were 61 and 230, respectively, for GCN- group. Heterogeneity test: *P * =0.02, *I *^2^ = 54%. In patients with mutant *KRAS*, the total events and patients were 7 and 44, respectively, for GCN + group, and were 2 and 97, respectively, for GCN- group. Heterogeneity test: *P* =0.005, *I*^2^ = 70%.

Based on the data from 10 studies [14,16,18,19,22,24-26,28,31], we further examined the association of *EGFR* GCN status with objective response in wild-type and mutant *KRAS* patients, respectively (Figure 3). Apparently, the difference in ORRs between GCN + and GCN- patients was much greater in wild-type than in mutant *KRAS* patients. Among patients with *KRAS* mutations, there was usually no difference between GCN + and GCN- patients. The only exception is the study of Moroni et al. (Figure 3), in which the sample size was quite small, and both patients in the GCN + group had *EGFR* amplification [14].

PFS data was reported in 15 studies (Table 3), of which 13 showed a trend of longer PFS in GCN + patients than in GCN- patients, although the difference was tested for significance in only ten studies and was statistically significant only in six of them. Two studies [21,29] reported hazard ratios for the comparison of the PFS of GCN + versus GCN- patients, which were 0.54 (95% CI: 0.32-0.93) and 0.82 (95% CI: 0.29-2.26), respectively, both favoring GCN + patients. Eight studies provided data on OS [15-18,20,28,31,32], and six of them reported a longer OS in GCN + than in GCN- patients, although the difference was statistically significant in only two studies.

**Table 3:** **The relation of *EGFR* gene copy number status with progression-free survival and overall survival**

**Study**	**Median PFS (mo)**	**HR**	** *P* **	**Median OS (mo)**	**HR**	** *P* **
	**GCN + vs GCN-**			**GCN + vs GCN-**		
Bengala 2009^25^	--	--	NS	--	--	NS
Campanella 2010^26^	--	0.54	0.03	--	--	--
Cappuzzo 2008^23^	6.6 vs 3.5*	--	0.02	11.3 vs 8.5*	--	0.8
Goncalves 2008^29^	4.1 vs 3.3	--	--	--	--	--
Italiano 2008^15^	--	--	0.70	--	--	0.82
Khambata-Ford 2007^30^	4.8 vs 2.0	--	--	--	--	--
Laurent-Puig 2009^16^	7.9 vs 6.5	--	0.280	19.7 vs 13.9	--	0.180
Mancuso 2008^32^	6.2 vs 3.2	--	0.003	--	--	--
Perrone 2009^24^	6 vs 5	--	--	--	--	--
Personeni 2008^33^	5.5 vs 4	--	0.25	10 vs 8.3	--	0.037
Razis 2008^34^	8.7 vs 6.4-6.9*	0.82	NS	--	--	--
Sartore-Bianchi 2007^17^	--	--	0.039	--	--	0.015
Sastre 2009^35^	4.9 vs 2.6	--	0.03	11.1	--	--
Scartozzi 2009^36^	7.7 vs 2.9	--	0.04	16 vs 9.5	--	0.2
Tol 2010^37^	9.5 vs 10.4	--	0.19	21.9 vs 22.0	--	0.65

PFS, progression-free survival; mo, month; GCN+, increased gene copy number; GCN-, non-increased gene copy number; HR, hazard ratio; OS, overall survival; --, not available; NS, not significant (without detailed numerical values).

* The original studies did not specify whether the number was “mean” or “median”.

### Publication bias

Because of the substantial heterogeneity among the included studies, we did not conduct the test for publication bias using funnel plot, for it would probably be misleading in this case [33,34].

## Discussion

This systematic review summarized the evidences on the predictive value of *EGFR* GCN + for clinical outcomes of mCRC treated with anti-EGFR MAbs. The data we collected showed that generally GCN + was associated with a better objective response, especially among patients with wild-type *KRAS*, which supports the notions that *KRAS* mutations are a strong predictor of non-response to the anti-EGFR MAbs treatment [11-13], and new biomarkers for the treatment would be primarily useful in *KRAS* wild-type patients [35].

However, the present systematic review was limited by the following factors. First, the majority of the included studies was retrospective in their nature, and thus might have suffered from some important bias. Second, there was significant heterogeneity among the studies, which precluded a clinically meaningful meta-analysis of the quantitative data. Third, although the PFS and OS were seemingly longer in GCN + than in GCN- patients, the data on these outcomes was relatively incomplete to convincingly support our conclusion on objective response.

More importantly, current evidences suggest that the clinical utility of *EGFR* GCN would be severely limited by two major problems. First, the scoring system of *EGFR* GCN has a high inter-laboratory variability, and none of the criteria used to define GCN + universally outperformed other criteria in terms of the discriminatory power. In some studies, the cutoff point for GCN + was identified by Receiver Operating Characteristics analysis, but frequently a cutoff value shown to have good sensitivity and specificity in one study performed less well in another. For example, in the study of Cappuzzo et al. [18], the cutoff point “gene copies/nucleus ≥ 2.92” categorized the patients into two groups in which the ORR were 33% (14/43) and 2% (1/42), respectively, with a sensitivity of 58.6% and a specificity of 93.3%. However, when the cutoff value was applied to the patients in the study of Personeni et al. [28], the corresponding sensitivity and specificity were only 56.0% and 75.8%, respectively. A standard cutoff value that can be used as a reference is yet to be established. Of note, even in studies that used the same criteria for GCN+, there was also significant variability in the difference of ORRs between GCN + and GCN- patients. For example, see the studies of Gevorgyan et al., Goncalves et al., Italiano et al. and Sastre et al. (Figure 2).

The criteria for *EGFR* gene amplification have been relatively consistent across studies [15,16,21,24], and patients with this molecular alteration generally had good response to anti-EGFR MAbs. In addition, it is readily identifiable by fluorescent in situ hybridization (FISH) assay. However, *EGFR* gene amplification proved to be a rare event, rendering it clinically less significant. Most patients defined as GCN + in the studies were actually harboring “polysomy” or “high polysomy”. Whether these statuses are equal to amplification in terms of their biological effects and especially the impact on the response to anti-EGFR MAbs remains unclear.

Second, the enumeration of *EGFR* GCN suffers from poor reproducibility. One reason for this is the within-tumor variation [36]. For example, the mean *EGFR* GCN of different sections within a tumor could be highly heterogeneous, leading to potential misclassification of *EGFR* GCN status in up to 39% of patients [28]. To a greater degree, the poor reproducibility is due to technical factors. For example, the thickness of tumor sections might affect the *EGFR* GCN detected, with thinner sections possibly responsible for a lower GCN cutoff value [18]. Notably, a recent international inter-laboratory reproducibility ring study [37] conducted by five “highly experienced molecular diagnostic centers” showed that even under standardized conditions, the results of FISH analysis, which was the most commonly used method the determine *EGFR* GCN, could still vary drastically from one laboratory to another. The low consensus rate was proposed to be related with such technical factors as the equipment used for the analyses, the skills necessary to perform enumeration of GCN, and the personnel difference in interpreting the pre-specified guidelines [37]. Although it is possible to enhance the consensus by intensive staff training in a research setting, it would be difficult to achieve this goal in routine practice. To date, scientifically validated and widely accepted protocol and guidelines for detecting *EGFR* GCN, which have been available for non-small cell lung cancer [38], remain to be developed for mCRC. This may partly explain why *EGFR* GCN has not yet been incorporated into clinical practice.

## Conclusions

Although increased *EGFR* GCN is generally associated with a better outcome of anti-EGFR MAbs treatment, especially among patients with wild-type *KRAS*, the clinical utility of this biomarker for selecting recipients of anti-EGFR MAbs would be severely limited by the heterogeneous scoring system and the poor reproducibility of *EGFR* GCN enumeration due to technical reasons.

## Methods

### Literature search

We performed a systematic search of PubMed, EMBASE, The Cochrane Library, Chinese Biomedical Literature Database, and Wanfang Data from inception to 22 November 2010. The detailed search strategy was described in the Additional file 1. Briefly, both the MeSH terms and various text words for CRC, MAbs and EGFR were used to identify relevant publications. The search was in the end limited to “human studies”. In the light of the results of our pilot search, we did not include the terms related to the biomarker (i.e. “gene copy number”) and concerned outcomes (e.g., “objective response” and “overall survival”) in the final search strategy so as to increase the search sensitivity. In addition to searching the above electronic databases, we also tried to identify eligible studies from the conference abstracts of American Society of Clinical Oncology and European Society of Medical Oncology via their official websites. All potentially relevant studies were retrieved and their references were scrutinized for further relevant publications.

### Study selection

All “potentially eligible” studies were reviewed independently and then agreed on their eligibility by two reviewers. Studies that met all of the following four criteria were considered eligible for this review: 1) patients: mCRC; 2) treatment: MAbs as monotherapy or in combination with other agents for treatment of any lines; 3) biomarker: *EGFR* GCN; and 4) outcomes: one or more of the following outcomes stratified by *EGFR* GCN status: objective response (the sum of complete response and partial response), PFS, and OS. Although PFS theoretically differs from time-to-progression, the two outcomes were often used interchangeably in existing clinical cancer research. Therefore, we did not distinguish them in this meta-analysis, but used PFS alone to denote either of the two. When the same patient population was used in more than one publication, only the one with most relevant data was included in this review. Disagreements between the two reviewers were resolved by discussion. Unsettled disagreements which were few were referred to the “third wise man” for final verdict.

### Data extraction

The following data were collected from each eligible study: first author’s name, year of publication, study design, total number of patients eligible to be included in this systematic review, *KRAS* mutation status, number of patients with increased *EGFR* GCN (GCN+) (stratified by *KRAS* mutation status, if applicable), number of patients with non-increased GCN (GCN-) (stratified by *KRAS* mutation status, if applicable), line of treatment, treatment regimen, response criteria, original location of tumor tissues used for analysis, method for *EGFR* GCN analysis, criteria for “GCN+”, objective response rate (ORR), PFS, OS, and hazard ratio for the comparison of PFS/OS. Data on ORR, PFS, and OS were extracted separately according to *EGFR* GCN status (further according to *KRAS* mutation status, if applicable).

With respect to the line of treatment, we pragmatically categorized a study as “1^st^-line” if ≥ 90% of the patients received 1^st^-line MAbs treatment. Similarly, a study was considered as “≥ 2^nd^-line” if ≥ 90% of the patients received 2^nd^-line or higher anti-EGFR MAbs treatment. All other studies were categorized as “mixed”. The same principle was applied to “treatment regimen” (monotherapy vs combined-therapy vs mixed) and “original location of tumor tissues used for analysis” (primary vs metastatic vs mixed).

If any key data (e.g. the number of patients responsive to anti-EGFR MAbs by *EGFR* GCN status) was absent in the original paper, authors were contacted by e-mail for relevant information.

### Statistical methods

The outcomes of interest included objective response, PFS, and OS. The impact of *EGFR* GCN status on objective response was measured by risk difference, which was the ORR of patients with GCN + subtracted by that of patients with GCN-. The association of *EGFR* GCN status with PFS or OS was denoted by hazard ratio. A hazard ratio equal to one means no difference between the compared groups. A hazard ratio less than one indicates that the risk for disease progression or death was lower in patients with GCN + than in those with GCN-, i.e. the PFS or OS of patients with GCN + was longer than that of patients with GCN-, and vice versa.

The statistical heterogeneity among studies was assessed by the Cochran’s *Q*-test [39,40] and the *I*^*2*^ statistic [40,41]. A *P* value ≤ 0.10 for the *Q*-test or an *I*^*2*^ > 50% was suggestive of substantial between-study heterogeneity. The clinical and methodological characteristics of the eligible studies were also examined to see if a quantitative synthesis of the collected data was appropriate. If not, then the data was summarized and presented in a descriptive manner; if yes, then the risk differences and hazard ratios respectively from different studies were combined by using a random-effects model (DerSimonian and Laird method) [41,42]. Further meta-analyses of risk difference and/or hazard ratio, stratified by *KRAS* status, would be performed wherever possible.

If appropriate and data allowed us to do so, pre-specified subgroup analyses were conducted to explore the source of the heterogeneity according to treatment regimen, line of treatment, response criteria, original location of tumor tissues used for analysis, method for *EGFR* GCN analysis, and the cutoff value for GCN+. Egger’s funnel plot was planned to be used to assess the possibility of publication bias as appropriate [43]. All statistical analyses were performed in RevMan 5.0.

## Abbreviations

CEP7, chromosome 7; EGFR, epidermal growth factor receptor; GCN, gene copy number; GCN+, increased epidermal growth factor receptor gene copy number; GCN-, non-increased epidermal growth factor receptor gene copy number; mCRC, metastatic colorectal cancer; MAbs, monoclonal antibodies; PFS, progression-free survival; ORR, objective response rate; OS, overall survival.

## Competing interests

The authors have declared no conflicts of interest.

## Authors’ contributions

ZYY, WXS, CM, and JLT designed the systematic review; ZYY, WXS, XFH, DYZ, XYW, YFH, JZC, and CM were involved in the literature search and study selection; ZYY, XFH, XYW, YFH, and CM extracted the data from eligible studies; ZYY and CM conducted the analysis; ZYY, WXS, DYZ, JZC, CM, and JLT were involved in the interpretation of the results. ZYY, WXS, CM, and JLT were responsible for the writing and critical revisions of the manuscript. All authors read and approved the final manuscript.

## Supplementary Material

Additional file 1 ** Detailed search strategy.** This document describes the search strategy in details. Click here for file

## References

[B1] GLOBOCAN Cancer fact sheet 2008 [http://globocan.iarc.fr/factsheets/cancers/colorectal.asp#INCIDENCE1]

[B2] Meyerhardt JA Mayer RJ Systemic therapy for colorectal cancer N Engl J Med 2005 352 476 487 10.1056/NEJMra040958 15689586

[B3] Sargent DJ Wieand HS Haller DG Disease-free survival versus overall survival as a primary end point for adjuvant colon cancer studies: individual patient data from 20,898 patients on 18 randomized trials J Clin Oncol 2005 23 8664 8670 10.1200/JCO.2005.01.6071 16260700

[B4] Bokemeyer C Bondarenko I Makhson A Fluorouracil, leucovorin, and oxaliplatin with and without cetuximab in the first-line treatment of metastatic colorectal cancer J Clin Oncol 2009 27 663 671 10.1200/JCO.2008.20.8397 19114683

[B5] Tol J Punt CJ Monoclonal antibodies in the treatment of metastatic colorectal cancer: a review Clin Ther 2010 32 437 453 10.1016/j.clinthera.2010.03.012 20399983

[B6] Van Cutsem E Köhne CH Hitre E Cetuximab and chemotherapy as initial treatment for metastatic colorectal cancer N Engl J Med 2009 360 1408 1417 10.1056/NEJMoa0805019 19339720

[B7] Sobrero AF Maurel J Fehrenbacher L EPIC: phase III trial of cetuximab plus irinotecan after fluoropyrimidine and oxaliplatin failure in patients with metastatic colorectal cancer J Clin Oncol 2008 26 2311 2319 10.1200/JCO.2007.13.1193 18390971

[B8] Peeters M Price TJ Cervantes A Randomized phase III study of panitumumab with fluorouracil, leucovorin, and irinotecan (FOLFIRI) compared with FOLFIRI alone as second-line treatment in patients with metastatic colorectal cancer J Clin Oncol 2010 28 4706 4713 10.1200/JCO.2009.27.6055 20921462

[B9] Douillard JY Siena S Cassidy J Randomized, phase III trial of panitumumab with infusional fluorouracil, leucovorin, and oxaliplatin (FOLFOX4) versus FOLFOX4 alone as first-line treatment in patients with previously untreated metastatic colorectal cancer: the PRIME study J Clin Oncol 2010 28 4697 4705 10.1200/JCO.2009.27.4860 20921465

[B10] Schrag D The Price Tag on Progress — Chemotherapy for Colorectal Cancer N Engl J Med 2004 351 317 319 10.1056/NEJMp048143 15269308

[B11] Dahabreh IJ Terasawa T Castaldi PJ Systematic review: Anti-epidermal growth factor receptor treatment effect modification by KRAS mutations in advanced colorectal cancer Ann Intern Med 2011 154 37 49 21200037 10.7326/0003-4819-154-1-201101040-00006

[B12] Linardou H Dahabreh IJ Kanaloupiti D Assessment of somatic k-RAS mutations as a mechanism associated with resistance to EGFR-targeted agents: a systematic review and metaanalysis of studies in advanced non-small-cell lung cancer and metastatic colorectal cancer Lancet Oncol 2008 9 962 972 10.1016/S1470-2045(08)70206-7 18804418

[B13] Qiu LX Mao C Zhang J Predictive and prognostic value of KRAS mutations in metastatic colorectal cancer patients treated with cetuximab: a meta-analysis of 22 studies Eur J Cancer 2010 46 2781 2787 10.1016/j.ejca.2010.05.022 20580219

[B14] Moroni M Veronese S Benvenuti S Gene copy number for epidermal growth factor receptor (EGFR) and clinical response to antiEGFR treatment in colorectal cancer: a cohort study Lancet Oncol 2005 6 279 286 10.1016/S1470-2045(05)70102-9 15863375

[B15] Italiano A Follana P Caroli FX Cetuximab shows activity in colorectal cancer patients with tumors for which FISH analysis does not detect an increase in EGFR gene copy number Ann Surg Oncol 2008 15 649 654 10.1245/s10434-007-9667-2 17987340

[B16] Laurent-Puig P Cayre A Manceau G Analysis of PTEN, BRAF, and EGFR status in determining benefit from cetuximab therapy in wild-type KRAS metastatic colon cancer J Clin Oncol 2009 27 5924 5930 10.1200/JCO.2008.21.6796 19884556

[B17] Sartore-Bianchi A Moroni M Veronese S Epidermal growth factor receptor gene copy number and clinical outcome of metastatic colorectal cancer treated with panitumumab J Clin Oncol 2007 25 3238 3245 10.1200/JCO.2007.11.5956 17664472

[B18] Cappuzzo F Finocchiaro G Rossi E EGFR FISH assay predicts for response to cetuximab in chemotherapy refractory colorectal cancer patients Ann Oncol 2008 19 717 723 17974556 10.1093/annonc/mdm492

[B19] Perrone F Lampis A Orsenigo M PI3KCA/PTEN deregulation contributes to impaired responses to cetuximab in metastatic colorectal cancer patients Ann Oncol 2009 20 84 90 18669866 10.1093/annonc/mdn541

[B20] Bengala C Bettelli S Fontana A EGFR gene copy number, KRAS and BRAF status, PTEN and AKT expression analysis in patients with metastatic colon cancer treated with anti-EGFR monoclonal antibodies ± chemotherapy [abstract] J Clin Oncol 2009 27 15055

[B21] Campanella C Mottolese M Cianciulli A Epidermal growth factor receptor gene copy number in 101 advanced colorectal cancer patients treated with chemotherapy plus cetuximab J Transl Med 2010 8 36 43 10.1186/1479-5876-8-36 20398370 PMC2867799

[B22] Frattini M Saletti P Romagnani E PTEN loss of expression predicts cetuximab efficacy in metastatic colorectal cancer patients Br J Cancer 2007 97 1139 1145 10.1038/sj.bjc.6604009 17940504 PMC2360431

[B23] Gevorgyan A Di Bartolomeo M Andreola S Epidermal Growth Factor Receptor (EGFr) status detection in correlation to objective response on cetuximab-based therapy in patients (pts) with advanced colorectal cancer (ACC) [abstract]. In: 2007 ASCO Annual Meeting Proceedings Part I J Clin Oncol 2007 25 18 21070

[B24] Goncalves A Esteyries S Taylor-Smedra B A polymorphism of EGFR extracellular domain is associated with progression free-survival in metastatic colorectal cancer patients receiving cetuximab-based treatment BMC Cancer 2008 8 169 179 10.1186/1471-2407-8-169 18544172 PMC2432064

[B25] Khambata-Ford S Garrett CR Meropol NJ Expression of epiregulin and amphiregulin and K-ras mutation status predict disease control in metastatic colorectal cancer patients treated with cetuximab J Clin Oncol 2007 25 3230 3237 10.1200/JCO.2006.10.5437 17664471

[B26] Lievre A Bachet JB Le Corre D KRAS mutation status is predictive of response to cetuximab therapy in colorectal cancer Cancer Res 2006 66 3992 3995 10.1158/0008-5472.CAN-06-0191 16618717

[B27] Mancuso A Leone A Vigna L EGFR, DCC, and K-RAS mutations as predictive factors for cetuximab sensitivity in metastatic colorectal cancer (mCRC) [abstract] J Clin Oncol 2008 26 20 4128

[B28] Personeni N Fieuws S Piessevaux H Clinical usefulness of EGFR gene copy number as a predictive marker in colorectal cancer patients treated with cetuximab:a fluorescent in situ hybridization study Clin Cancer Res 2008 14 5869 5876 10.1158/1078-0432.CCR-08-0449 18794099

[B29] Razis E Briasoulis E Vrettou E Potential value of PTEN in predicting cetuximab response in colorectal cancer:an exploratory study BMC Cancer 2008 8 234 243 10.1186/1471-2407-8-234 18700047 PMC2527615

[B30] Sastre J Aranda E Grávalos C First-line single-agent cetuximab in elderly patients with metastatic colorectal cancer. A phase II clinical and molecular study of the Spanish group for digestive tumor therapy (TTD) Crit Rev Oncol Hematol 2011 77 78 84 Epub 2009 Dec 29 10.1016/j.critrevonc.2009.11.005 20042346

[B31] Scartozzi M Bearzi I Mandolesi A Epidermal Growth Factor Receptor (EGFR) gene copy number (GCN) correlates with clinical activity of irinotecan-cetuximab in K-RAS wild-type colorectal cancer:a fluorescence in situ (FISH) and chromogenic in situ hybridization (CISH) analysis BMC Cancer 2009 9 303 311 10.1186/1471-2407-9-303 19712476 PMC3087339

[B32] Tol J Dijkstra JR Klomp M Markers for EGFR pathway activation as predictor of outcome in metastatic colorectal cancer patients treated with or without cetuximab Eur J Cancer 2010 46 1997 2009 10.1016/j.ejca.2010.03.036 20413299

[B33] Lau J Ioannidis JP Terrin N Schmid CH Olkin I The case of the misleading funnel plot BMJ 2006 333 597 600 10.1136/bmj.333.7568.597 16974018 PMC1570006

[B34] Tang JL Liu JL Misleading funnel plot for detection of bias in meta-analysis J Clin Epidemiol 2000 53 477 84 10.1016/S0895-4356(99)00204-8 10812319

[B35] Mao C Yang ZY Hu XF PIK3CA exon 20 mutations as a potential biomarker for resistance to anti-EGFR monoclonal antibodies in KRAS wild-type metastatic colorectal cancer:a systematic review and meta-analysis Ann Oncol 2012 23 1518 1525 10.1093/annonc/mdr464 22039088

[B36] Ooi A Takehana T Li X Protein overexpression and gene amplification of HER-2 and EGFR in colorectal cancers:an immunohistochemical and fluorescent in situ hybridization study Mod Pathol 2004 17 895 904 10.1038/modpathol.3800137 15143334

[B37] Sartore-Bianchi A Fieuws S Veronese S Standardisation of EGFR FISH in colorectal cancer:results of an international interlaboratory reproducibility ring study J Clin Pathol 2012 65 218 223 10.1136/jclinpath-2011-200353 22130903 PMC4612522

[B38] Varella-Garcia M Diebold J Eberhard DA EGFR fluorescence in situ hybridisation assay:guidelines for application to non-small-cell lung cancer J Clin Pathol 2009 62 970 977 10.1136/jcp.2009.066548 19861557 PMC2771853

[B39] Cochran WG The combination of estimates from different experiments Biometrics 1954 10 101 129 10.2307/3001666

[B40] Higgins JPT Thompson SG Deeks JJ Altman DG Measuring inconsistency in meta-analyses BMJ 2003 327 557 560 10.1136/bmj.327.7414.557 12958120 PMC192859

[B41] Deeks JJ Higgins JPT Altman DG Analysing and presenting results 2006 In: Higgins JPT, Green S, editors. Cochrane Handbook for Systematic Reviews of Interventions 4.2.6 [updated September 2006], Section 8, Chichester, UK: John Wiley & Sons, Ltd 4

[B42] DerSimonian R Laird N Meta-analysis in clinical trials Control Clin Trials 1986 7 177 188 10.1016/0197-2456(86)90046-2 3802833

[B43] Egger M Smith DG Schneider M Minder C Bias in meta-analysis detected by a simple, graphical test BMJ 1997 315 629 634 10.1136/bmj.315.7109.629 9310563 PMC2127453

