# Supplementary material for: EGFR gene copy number as a predictive biomarker for the treatment of metastatic colorectal cancer with anti-EGFR monoclonal antibodies: a meta-analysis
Source: J Hematol Oncol. 2012 Aug 16;5:52. doi: 10.1186/1756-8722-5-52 (PMC3447654; doi:10.1186/1756-8722-5-52)
Supplement: Additional file 1 — Detailed search strategy. This document describes the search strategy in details. [file 1756-8722-5-52-S1.pdf]

Additional file 1: Detailed search strategy

1. PubMed search

Search	Most Recent Queries	Result
#16	Search **#14 NOT #15**	403
#15	Search **#13** Limits: **Humans, Editorial, Letter, Review**	421
#14	Search **#13** Limits: **Humans**	824
#13	Search **#10 AND #11 AND #12**	924
#12	Search **#7 OR #8 OR #9**	32105
#11	Search **#3 OR #4 OR #5 OR #6**	217900
#10	Search **#1 OR #2**	142068
#9	Search **"Genes, erbB-1"[Mesh]**	559
#8	Search **"epidermal growth factor receptor" OR "egf receptor" OR egfr OR "erbb-1" OR "her1 in humans"**	32105
#7	Search **"Receptor, Epidermal Growth Factor"[Mesh]**	20976
#6	Search **"monoclonal antibody" OR "monoclonal antibodies" OR mab$ OR mcab$ OR moab$ OR cetuximab OR erbitux OR c225 OR "c-225" OR panitumumab OR "abx-egf" OR vectibix**	216836
#5	Search **"panitumumab "[Substance Name]**	250
#4	Search **"cetuximab "[Substance Name]**	1432
#3	Search **"Antibodies, Monoclonal"[Mesh]**	150573
#2	Search **"colon cancer" OR "colon carcinoma" OR "rectal cancer" OR "rectal carcinoma" OR "rectum cancer" OR "rectum carcinoma" OR "bowel cancer" OR "bowel carcinoma" OR "colorectal cancer" OR "colorectal carcinoma" OR CRC**	84749
#1	Search **"Colorectal Neoplasms"[Mesh]**	119565

2. EMBASE search

#	Searches	Results
1	colon cancer/	27731
2	rectum cancer/	11201
3	("colon cancer" or "colon carcinoma" or "rectal cancer" or "rectal carcinoma" or "rectum cancer" or "rectum carcinoma" or "bowel cancer" or "bowel carcinoma" or "colorectal cancer" or "colorectal carcinoma" or CRC).af.	145807
4	monoclonal antibody/	148815
5	cetuximab/	8796
6	panitumumab/	2259
7	("monoclonal antibody" or "monoclonal antibodies" or mab? or mcab? or moab? or cetuximab or erbitux or c225 or "c-225" or panitumumab or "abx-egf" or vectibix).af.	234299
8	epidermal growth factor receptor/	27739
9	("epidermal growth factor receptor" or "egf receptor" or egfr or "erbb-1" or "her1 in humans").af.	51583
10	1 or 2 or 3	145807
11	4 or 5 or 6 or 7	234299
12	8 or 9	51583
13	10 and 11 and 12	2421
14	(editorial or letter or "review").pt.	2724557
15	13 and 14	1168
16	13 not 15	1253
17	limit 16 to human	1043

3. The Cochrane Library search

ID	Search	Hits
#1	MeSH descriptor **Colorectal Neoplasms** explode all trees	4011
#2	"colon cancer" OR "colon carcinoma" OR "rectal cancer" OR "rectal carcinoma" OR "rectum cancer" OR "rectum carcinoma" OR "bowel cancer" OR "bowel carcinoma" OR "colorectal cancer" OR "colorectal carcinoma" OR CRC or "colon cancer" OR "colon carcinoma" OR "rectal cancer" OR "rectal carcinoma" OR "rectum cancer" OR "rectum carcinoma" OR "bowel cancer" OR "bowel carcinoma" OR "colorectal cancer" OR "colorectal carcinoma" OR CRC:ti,ab,kw	5506
#3	MeSH descriptor **Antibodies, Monoclonal** explode all trees	3117
#4	"monoclonal antibody" OR "monoclonal antibodies" OR mab OR mcab OR moab OR cetuximab OR erbitux OR c225 OR "c-225" OR panitumumab OR "abx-egf" OR vectibix or "monoclonal antibody" OR "monoclonal antibodies" OR mab OR mcab OR moab OR cetuximab OR erbitux OR c225 OR "c-225" OR panitumumab OR "abx-egf" OR vectibix:ti,ab,kw	2054
#5	MeSH descriptor **Receptor, Epidermal Growth Factor** explode all trees	191
#6	"epidermal growth factor receptor" OR "egf receptor" OR egfr OR "erbb-1" OR "her1 in humans" or "epidermal growth factor receptor" OR "egf receptor" OR egfr OR "erbb-1" OR "her1 in humans":ti,ab,kw	511
#7	MeSH descriptor **Genes, erbB-1** explode all trees	6
#8	((#1 OR #2) AND (#3 OR #4) AND (#5 OR #6 OR #7))	57

4. Chinese Biomedical Literature Database search

序号	命中文献数	检索表达式
17	50	(#12) not (#16)
16	19	#15 or #14 or #13
15	0	#10 and #9 and #8 -限定:译文; 人类
14	0	#10 and #9 and #8 -限定:讲座; 人类
13	19	#10 and #9 and #8 -限定:综述; 人类
12	69	#10 and #9 and #8 -限定:人类
11	77	#10 and #9 and #8
10	3820	#7 or #6 or #5
9	25939	#4 or #3
8	42486	#2 or #1
7	125	主题词:基因, erbB-1/全部树/全部副主题词
6	3803	全部字段:表皮生长因子 AND 受体
5	2653	主题词:受体, 表皮生长因子/全部树/全部副主题词
4	23921	全部字段:单克隆抗体 OR 单抗 OR 西妥昔 OR 爱必妥 OR 艾比特思 OR c225 OR "c-225" OR 帕尼 OR 维克替比
3	15895	主题词:抗体, 单克隆/全部树/全部副主题词
2	40375	全部字段:大肠癌 OR 结肠癌 OR 直肠癌
1	13034	主题词:结肠肿瘤/全部树/全部副主题词

5. Wanfang Data search

(大肠癌 OR 结肠癌 OR 直肠癌) AND 转移 AND (单克隆抗体 OR 单抗 OR 西妥昔 OR 爱必妥 OR 艾比特思 OR c225 OR "c-225" OR 帕尼 OR 维克替比) AND (表皮生长因子 AND 受体)

6. ASCO search

Website: http://www.asco.org/ASCOv2/Meetings/Abstracts

Selected meetings:
2007 Gastrointestinal Cancers Symposium
2007 ASCO Annual Meeting
2008 Gastrointestinal Cancers Symposium
2008 ASCO Annual Meeting
2008 Molecular Markers
2009 Gastrointestinal Cancers Symposium
2009 ASCO Annual Meeting
2010 Molecular Markers
2010 ASCO Annual Meeting
2010 Gastrointestinal Cancers Symposium

7. ESMO search

Website: http://www.esmo.org/education/abstracts-and-virtual-meetings.html

Selected meetings:

Part 1 Virtual meetings:

1. 2002 27th ESMO Congress, Nice
2. 2003 ESMO Scientific & Educational Conference, Edinburgh
3. 2004 29th ESMO Congress, Vienna
4. 2005 ESMO Scientific & Educational Conference, Budapest
5. 2006 31st ESMO Congress, Istanbul
6. 2007 ESMO Conference Lugano (ECLU)
7. 2008 33rd ESMO Congress, Stockholm
8. 2008 ESMO Conference Lugano (ECLU)
9. 2009 7th International Symposium on Targeted Anticancer Therapies
10. 2009 11th World Congress on Gastrointestinal Cancer
11. 2009 ECCO 15 and 34th ESMO Multidisciplinary Congress
12. 2010 8th International Symposium on Targeted Anticancer Therapies
13. 2010 12th World Congress on Gastrointestinal Cancer
14. 2010 35th ESMO Congress, Milan

Part 2 Other scientific meeting reports:

1. 2007 8th SIOG meeting, Madrid
2. 2007 ASCO Annual Meeting, Chicago
3. 2007 ECCO 14, Barcelona
4. 2008 ASCO Annual Meeting Chicago
5. 2008 BDA 9th International Symposium, Munich
6. 2009 10th Meeting of the International Society of Geriatric Oncology
7. 2009 ASCO Annual Meeting Orlando
